# Sea Voyaging

**Published:** 1884-04

**Authors:** 


### SEA VOYAGES.

WE ARE so accustomed to recommend a sea voyage to a patient whom we are not able otherwise to benefit, that it has become a therapeutic routine, and we rarely hesitate to consider the appropriateness of our recommendations. In the *Lancet* of February 23, Dr. Farquhar publishes a paper on Sea Change, the Effects of Voyaging, with Cases. He remarks on the paucity of information on this subject, Dr. Faber being the only observer who had gone to work systematically. Dr. Farquhar's observations on a voyage last winter, up the Mediterranean, led him to the belief that many unsuitable cases are sent a voyaging by medical men, and in the event of such a rigorous winter as that of last year, even in Italy and Greece, the disappointment to invalids in search of health must be great.

He then gives details of the various cases that came under his care and observation. The most pronounced benefit was obtained by most of the phthisical cases, but some left the steamer before the tonic and stimulating action of the sea air had made itself felt, unnerved by the hardships endured in a storm experienced on leaving the Mersey. Voyagers suffering from the effects of overwork and mental strain did well; but "specific" cases showed the most marvellous results, complete recovery being the rule. Dyspeptics did not do well, nor subjects of skin disease of a nervous origin. To sum up, his experience in this voyage led him to believe that no diseased condition where the nervous, irritable element was present, had much chance of benefit; the sea air stimulus was in the end too powerful through its constancy. Such cases should land after the first beneficial stimulation, which generally occurred within the first three or four weeks of the voyage.

### PERPETUAL INJUNCTION.

In the U. S. Circuit Court in Maryland, it was, on the 10th of March, 1884, adjudged and decreed that a perpetual injunction be issued against Louis E. Wetter, and eighteen others, restraining them from imitating the labels of the Rumford Chemical Works, manufacturers of Horsford's Baking Powder, and also from using their old bottles.

The defendants were required to bring into court all fraudulent labels and all imitation powder, for destruction. It was decreed that the Rumford Chemical Works be entitled to receive the profits which have been diverted from it by reason of the infringement, and the defendants were ordered to pay all costs. Thus is another victory scored for the Rumford Chemical Works who, not long since, caused several parties to be heavily fined for violating the injunction of the Supreme Court restraining all persons from offering for sale "Acid Phosphate" (so called) in any package which shall be a substantial or colorable imitation of Horsford's Acid Phosphate.

